# Notice of Leidy’s Anatomy

**Published:** 1860-11

**Authors:** 


*An Elementary Treatise on Human Anatomy.* By JOSEPH LEIDY, Professor of Anatomy in the University of Pensylvania, &c., &c.

We are in receipt of the above work just published by J. B. Lippincott & Co., Philadelphia. It contains over 600 pages, with three hundred and ninety-two illustrations.—These are the finest wood cuts that we have ever examined. The book, as the title imports, is intended for those not advanced in the study of Anatomy; and, from the hasty perusal we have been able to give it, is admirably adapted to the wants of medical students. In the preface the author says:

"Much of the difficulty of which we hear constant complaint, in the acquisition and retention of anatomical knowledge, arises from an excessive, and, in some other respects, objectionable nomenclature. Not only has the naming of comparatively unimportant parts been carried to an extreme, but, in numerous instances, the same parts are designated by a multitude of names, which are indiscriminately used by different writers.

"The nomenclature of anatomy has been founded on no particular system; the names having been chosen, according to the fancy of anatomists, from the shape, function, or supposed resemblance of the part, or in commemoration of the original investigator."

The work abounds with the most beautiful wood cuts from the magnified drawings of the structure of the various tissues, as well as accurate illustrations of the form and position of the different parts of the body. The arrangement of the work in this particular is admirable. Each plate is placed on the same page, and alongside of the description of the part represented, so that the description and representation are both under the eye in close connection.

We are decidedly pleased with the manner in which the technical terms or names of parts are brought prominently before the reader by heavier type; and with the advantage afforded the beginner in pronouncing them correctly, by the marking of accents.

As the author has stated in his preface, those terms are used which are most common and expressive, while the synonyms are given in foot-notes and referred to by their proper numbers.

Taken altogether, we think the book a useful one, and feel confident that the student of medicine will find it greatly to his interest to avail himself of the advantages of its perusal. Our time and space do not admit of a more extended notice now.

